# How *Mycobacterium tuberculosis* Subverts Innate and Adaptive Immunity and Their Crosstalk: Implications for Vaccine Design

**DOI:** 10.3390/vaccines14050414

**Published:** 2026-05-02

**Authors:** G V R Krishna Prasad, Jennifer A. Philips

**Affiliations:** 1 Division of Infectious Diseases, Department of Medicine, Washington University School of Medicine, St. Louis, MO 63110, USA; 2 Department of Molecular Microbiology, Washington University School of Medicine, St. Louis, MO 63110, USA

**Keywords:** tuberculosis, immune response, bacterial evasion, macrophages, T cells, heterogeneity, autophagy, metabolism, vaccine efficacy

## Abstract

Globally, *Mycobacterium tuberculosis (Mtb)* remains the leading cause of death from a single infectious agent. The only licensed vaccine, Bacillus Calmette–Guérin (BCG), was developed over a century ago and does not provide consistent protection against pulmonary tuberculosis (TB). Efforts to develop more effective vaccines are hindered by an incomplete understanding of the correlates of protection and by the pathogen’s sophisticated immune-evasion strategies. *Mtb* systematically undermines host defenses, reprograms host cell biology, and interferes with cell–cell communication to establish a permissive niche and sustain chronic infection. An effective vaccine must elicit immune responses capable of overcoming these bacterial strategies across diverse host and pathogen backgrounds. Traditional approaches focused on boosting T cell responses have proven inadequate. In this review, we summarize innate and adaptive immune mechanisms that contain *Mtb*, examine how bacterial immune subversion and host–pathogen heterogeneity complicate vaccine design, and highlight emerging concepts and strategies to guide TB vaccine development.

## 1. Introduction

Tuberculosis (TB), caused by *Mycobacterium tuberculosis* (*Mtb*), has been the leading cause of death due to an infectious pathogen for decades. The year 2023 recorded the highest number of newly diagnosed TB patients since the World Health Organization (WHO) began global TB surveillance in 1995, with an estimated 8.2 million cases and 1.25 million TB-related fatalities [1]. Most infected individuals either clear the pathogen or live asymptomatically with latent TB infection (LTBI), but approximately 5–10% go on to develop active disease [2,3]. The Bacillus Calmette–Guérin (BCG) vaccine, developed over 100 years ago using attenuated *Mycobacterium bovis*, is the only available vaccine. Several epidemiological studies, efficacy trials, and meta-analyses have shown that the BCG vaccine protects against disseminated TB in children but induces minimal and variable protection in adults [4,5]. With rising cases of drug-resistant TB, the variable efficacy of BCG, and the high prevalence of TB worldwide, there is an urgent need to develop an efficacious vaccine, but this has continued to elude investigators, and there remain numerous challenges. A vaccine for TB could either protect against infection (a preventive vaccine) or inhibit progression to active disease (a therapeutic vaccine). It should elicit long-lasting immunity and be safe to administer to diverse populations, including patients with HIV. However, clinical development of a vaccine is hampered by the lack of a diagnostic test to identify individuals who harbor viable bacteria and are therefore at risk of developing active disease. Also, we currently lack a clear understanding of the immune correlates of protection, and it has been difficult to define which *Mtb* antigens should be included in a vaccine. The coevolution between *Mtb* and humans for nearly 70,000 years has allowed the bacilli to develop a sophisticated repertoire of virulence factors that transform host phagocytic cells into a replicative niche and evade host immune defenses [6,7]. The ability of a vaccine to protect individuals depends on whether the immune responses it generates overcome *Mtb*’s immune evasion strategies. Additional impediments to an effective vaccine include heterogeneity in the bacterial population and in immune responses among individuals. In this review, we discuss how the host immune system responds to and defends against *Mtb*, and how bacterial immune subversion mechanisms and the heterogeneity of bacteria and hosts pose challenges for vaccine development, as well as potential strategies to improve TB vaccines.

## 2. Overview of Innate Immune Response

**Figure 1:**
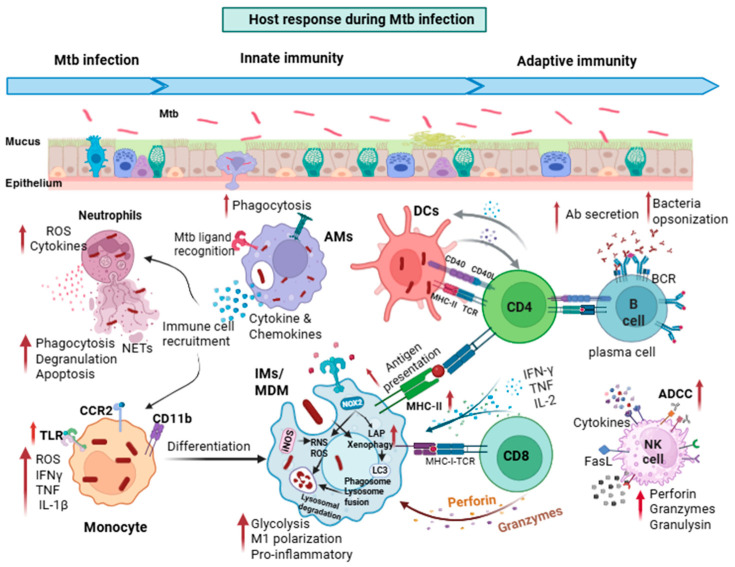
**Immune cell responses against *Mtb***. Schematic of the host cellular response to *Mtb*. *Mtb* interacts with the airway epithelium. The bacilli are phagocytosed by AMs, which then migrate to the interstitium and secrete cytokines and chemokines to recruit neutrophils, monocytes, IMs, and DCs. AMs undergo cell death and release replicating *Mtb*. Neutrophils phagocytose or trap *Mtb* in NETs, release ROS, and undergo degranulation to release antimicrobial peptides. CCR2-expressing monocytes infiltrate into the lungs, recognize *Mtb* ligands using PRRs, secrete pro-inflammatory cytokines, and differentiate into macrophages or DCs. IMs/MDMs phagocytose *Mtb* released by dead infected cells. Xenophagy and LAP pathways contribute to the clearance of bacteria. Macrophages undergo metabolic reprogramming and produce RNS and ROS. Macrophages process *Mtb* antigens in phagolysosomes and present them to T cells through MHCII. DCs transport *Mtb* to draining lymph nodes and activate naïve T cells. Activated T cells migrate to the lungs, producing IFNγ and TNF, which further activate macrophage anti-mycobacterial activity and the killing of infected cells. B cells differentiate into antibody-producing plasma cells. Antibodies opsonize *Mtb* or infected cells. NK cells employ Fc receptors to recognize opsonized *Mtb* or infected cells and contribute to antibody-dependent cell cytotoxicity. Created in BioRender. Mittal, E. (2026) https://BioRender.com/92lgdaj. **Abbreviations:** *Mtb*—Mycobacterium tuberculosis, AMs—alveolar macrophages, DCs—dendritic cells, IMs—interstitial macrophages, MDMs—monocyte-derived macrophages, NK—natural killer cells, ROS—reactive oxygen species, RNS—reactive nitrogen species, NETs—neutrophil extracellular traps, iNOS—inducible nitric oxide synthase, NOX2—NADPH oxidase, IFN—interferon, IL—interleukin, PRRs—pattern recognition receptors. LAP—LC3-associated phagocytosis.

TB infection occurs upon inhalation of bacteria, with one to five bacilli sufficient to establish infection, demonstrating the exquisite ability of *Mtb* to undermine immunity. The early responses of the innate and adaptive immune systems determine bacterial fate, which can range from pathogen elimination to uncontrolled replication and dissemination. Innate immune cells employ a range of coordinated defense mechanisms (Figure 1). In the lungs, *Mtb* interacts with alveolar epithelial cells (AECs). AECs can provide structural defense, produce antimicrobial molecules, and communicate with innate immune cells by secreting cytokines and chemokines. In addition, they may provide a niche for *Mtb* and shape bacterial phenotypes, although this is a relatively poorly understood aspect of the infection [8,9]. In mice, the first cells that are productively infected are alveolar macrophages (AMs) [10]. Initially, AMs mount a host-protective antioxidant response through nuclear factor erythroid 2-related factor 2 (NRF2) and heme oxygenase-1 (Hmox1), a response that is ineffective in antibacterial defense [11,12,13]. In AMs, *Mtb* has abundant access to iron and fatty acids to support its growth [14,15]. Infected AMs are thought to migrate into the lung interstitium, driven by IL-1β signaling and the *Mtb* type VII secretion system ESX-1 [10]. After the first week of infection, additional immune cells are recruited, including neutrophils, monocytes, interstitial macrophages (IMs), dendritic cells (DCs), and B and T cells, which together form the characteristic granuloma. Neutrophils phagocytose *Mtb* and deploy reactive oxygen species (ROS) and neutrophil extracellular traps (NETs), but these mechanisms fail to eradicate the bacilli, which frequently survive within and around neutrophils. *Mtb* appears to exploit neutrophils as a replicative niche and to induce necrosis to facilitate bacterial dissemination [16,17,18]. Interstitial macrophages (IMs) can be tissue-resident or monocyte-derived; they are M1-like, pro-inflammatory cells with metabolic and functional characteristics that are distinct from AMs. IMs can restrict *Mtb* growth through a variety of mechanisms, including limiting iron availability, activating autophagy, driving more protective forms of cell death, and generating oxidative stress [19]. They also produce cytokines such as IL-1β, IL-6, and TNF to recruit immune cells and maintain the inflammatory tissue environment necessary to control *Mtb* infection. As the infection progresses, recruited IMs and DCs become the predominant infected cell subsets, outnumbering infected AMs [20,21]. *Mtb*-infected IMs and DCs migrate to the draining lymph nodes, where they release soluble, unprocessed *Mtb* antigens for uptake by uninfected conventional DCs, which then present antigens to naïve T cells, thereby activating the adaptive immune system [22,23,24].

Mechanisms of Defense

Whether the innate immune system protects some individuals so that they clear the infection without developing an adaptive immune response is unknown. At least in animal models, the innate immune response has limited efficacy in controlling bacterial expansion in the lungs. Key innate immune mechanisms that can confer some degree of protection are discussed below.

### 2.1. The Mucosal Barrier

As a respiratory pathogen, *Mtb* must contend with innate airway defenses including glycosylated mucins, defensins, immunoglobulins, lysozymes, and surfactant proteins, each with unique antibacterial functions. Mucins form a viscoelastic gel that can prevent mycobacteria from interacting with epithelial cells [25]. Defensins may directly kill mycobacteria by disrupting the envelope [26,27]. Secretory immunoglobulins may opsonize the bacilli, block *Mtb* adherence to the mucosal epithelium, and target the bacteria for clearance by resident innate phagocytes to prevent initial infection [28]. Surfactant proteins affect pathogen uptake and intracellular survival [29,30]. However, the extent to which these airway defenses protect against *Mtb* infection is not well understood.

### 2.2. Pathogen Sensing

Innate immune cells possess a repertoire of surface and intracellular receptors that recognize the conserved molecular structures of pathogens. These receptors include pathogen recognition receptors (PRRs), such as Toll-like receptors (TLRs), nucleotide-binding oligomerization domain (NOD)-like receptors (NLRs), C-type lectin receptors (CLRs), scavenger receptors, Fc receptors, and complement receptors. TLRs recognize diverse mycobacterial molecules, including acylated lipoproteins, lipoarabinomannan (LAM), and lipomannan (LM), as well as mycobacterial RNA, CpG-rich DNA, and heat shock proteins [31,32,33,34]. CLRs recognize mycolic acid, glycolipids, mannose-capped LAM (ManLAM), and trehalose dimycolate (TDM) [35,36], whereas intracellular NLRs are activated by *Mtb* ligands such as muramyl dipeptide [37,38]. In addition, cytosolic mycobacterial DNA activates the cGAS-STING pathway [39]. The role of immune receptors in *Mtb* pathogenesis has been extensively reviewed [40,41]. Ligand recognition promotes bacterial internalization and activates downstream signaling cascades, most notably NF-kB, MAPK, cGAS-STING, MyD88-dependent mechanisms, and inflammasomes. These pathways modulate immune cell functions like cytokine production, cell death, autophagy, phagocytosis, oxidative burst, and pyroptosis, thereby stimulating immunity and promoting pathogen clearance. To limit adverse immunopathology from excessive inflammation, lung immune cells also produce anti-inflammatory cytokines, including IL-10, IL-13, TGF-β, and IL-4. Thus, effective defense against *Mtb* relies on a balance between inflammation-driven protective responses that control bacterial growth and anti-inflammatory responses that prevent tissue damage.

### 2.3. Cell Death

Cell death is vital for maintaining tissue homeostasis and shapes innate immunity during *Mtb* infection. Multiple cell death programs, including apoptosis and distinct forms of regulated necrosis, are involved in *Mtb* pathogenesis. Apoptosis of *Mtb*-infected macrophages is generally associated with protection; the uptake of *Mtb*-infected apoptotic cells by uninfected phagocytes via efferocytosis leads to efficient killing of *Mtb* [42,43,44]. Efferocytosis-mediated bacterial killing depends on the recruitment and activation of the NADPH oxidase complex and on ROS generation within *Mtb*-containing apoptotic bodies. Apoptosis can be initiated by several pathways during *Mtb* infection, including TNF-driven death-receptor signaling and intrinsic mitochondrial stress, leading to the activation of MAPK pathways and the phosphorylation of pro-apoptotic BH3-only proteins and *Mtb*-induced mitochondrial outer-membrane permeabilization (MOMP), causing cytochrome C release, cytosolic apoptosome formation, executioner caspase 3/9 activation, and cell death [42]. In contrast, necrotic cell death is characterized by nuclear swelling and loss of plasma membrane integrity, releasing pro-inflammatory cytoplasmic content and intracellular *Mtb* into the extracellular space. This process promotes *Mtb* survival, dissemination, and tissue damage, and is detrimental to the host [42]. Ferroptosis is another major regulated necrotic pathway in *Mtb*-infected macrophages that benefits bacteria [42]. Overall, the balance between apoptotic and necrotic cell death pathways during *Mtb* infection influences the fate of *Mtb* and disease progression.

### 2.4. Reactive Oxygen and Nitrogen Species

Professional phagocytes utilize ROS and reactive nitrogen species (RNS) as primary defense mechanisms. ROS are either produced by the NADPH oxidase complex or derived from the mitochondria. ROS can damage *Mtb* DNA, proteins, and lipids, although *Mtb* is relatively resistant to phox-derived ROS. Observations in both human TB patients and mouse models support a modest role of ROS in *Mtb* control. In humans, mutations that disrupt NADPH oxidase (NOX2) expression or function (chronic granulomatous disease) increase susceptibility to mycobacterial infection, particularly with BCG [45]. NOX2-deficient mice typically show only modest increases in *Mtb* burden, but they exhibit a hyperinflammatory response due to excess IL-1β production and neutrophil influx into the lungs [46], indicating that the phagocyte oxidase constrains immunopathology. In contrast, NADPH oxidase-deficient mice infected with BCG develop severe lung injury, high bacterial burden, and increased mortality [47,48]. Interestingly, in mice, the aggregation of platelets with macrophages and PMNs restricts NOX2-mediated ROS production, resulting in an increased *Mtb* load and adverse lung pathology [49]. When activated pharmacologically, for example, by inhibiting fatty acid oxidation, mitochondrial ROS can enhance intracellular control of *Mtb* [50]. Overall, these data suggest that ROS make a limited contribution to directly killing *Mtb*, which can be explained by the ability of the bacilli to inhibit and detoxify ROS. Evidence suggests that NO is also protective. Human granulomas contain iNOS, endothelial NOS, and nitrotyrosine in areas that are enriched with inflammatory epithelioid macrophages, indicating iNOS activation and NO production during TB infection [51]. Mice genetically lacking inducible NO production or treated with iNOS inhibitors are highly susceptible to *Mtb* infection [52,53]. NO plays a role in *Mtb* control in mouse macrophages (but not human monocyte-derived macrophages) ex vivo [54]. Interestingly, mice deficient in both phagocyte oxidase and NOS2 are far more susceptible to infections than those deficient in either enzyme alone, indicating that RNS and ROS provide partially redundant protection [55].

### 2.5. Autophagy

Innate immune cells employ autophagy as a defense mechanism to target intracellular *Mtb* for lysosomal killing and to modulate the inflammatory response. In canonical autophagy, intracellular bacteria are sequestered into double-membrane compartments called autophagosomes, which subsequently fuse with lysosomes, where acidic pH, hydrolytic enzymes, and antimicrobial peptides can restrict *Mtb* [56]. During *Mtb* infection, ROS production also triggers a non-canonical autophagy pathway called LC3-associated phagocytosis (LAP) in which *Mtb* elimination occurs in LC3-coated, single-membrane phagosomes that rely on a subset of proteins involved in canonical autophagy [56]. PRRs, inflammatory cytokines, vitamin D3, and numerous small molecules have been reported to activate autophagy and thereby enhance *Mtb* control in macrophages ex vivo [57]. For example, primary human macrophages treated with carbamazepine or valproic acid enhance autophagy and can reduce intracellular *Mtb* burden [58]. Autophagy also limits excessive production of inflammatory cytokines and neutrophil recruitment in the lungs, preventing extensive tissue damage [59,60]. In mouse models, autophagy and related LC3-dependent pathways make a modest contribution to direct bacterial control and are important for restraining destructive immunopathologic responses [61,62,63,64]. Overall, key innate immune defenses, including mucosal barriers, programmed cell death, ROS, NOS, and autophagy, confer only partial protection against *Mtb* because the bacilli successfully evade and subvert these pathways.

The innate immune system is crucial for establishing initial defense against *Mtb* and also for shaping subsequent host–pathogen interactions. By subverting innate defenses, including impairing autophagy and promoting necrosis over apoptosis, the bacilli not only impair the host’s to clear the acute infection, but also undermine essential immune crosstalk necessary for activating adaptive immunity for long-term protection.

## 3. Overview of Adaptive Immune Response to *Mtb*

The onset of adaptive immunity marks the transition to more robust control over *Mtb*. Adaptive immunity typically takes 2–6 weeks to develop after *Mtb* infection.

### 3.1. T Cell-Mediated Protection

Adaptive immunity is initiated when *Mtb*-infected or antigen-bearing DCs migrate to the lymph node and present the *Mtb* antigens to naïve T cells. Upon antigen recognition, T cells are activated, proliferate, and migrate to the lung parenchyma, where they are critical for arresting mycobacterial growth and preventing further dissemination. CD4 T cells are considered the cornerstone of immunity against *Mtb*. In people with HIV (PWH), CD4 T cell numbers inversely correlate with susceptibility to TB infection [65]. In mice, depletion of CD4+ T cells causes higher bacterial load, increased extrapulmonary dissemination, and reduced survival [66,67]. In non-human primates (NHPs), CD4 T cell depletion, whether by using the anti-CD4 antibody or upon simian immunodeficiency virus infection, accelerates TB disease progression [68,69]. Beyond restricting *Mtb* growth, CD4 T cells limit necrotic lesion formation, excessive neutrophil infiltration, and tissue damage. Effector CD4 T cells mediate protective responses against *Mtb* infection through direct contact with infected macrophages and by cytokine secretion. In mice, direct interaction of CD4 T cells with infected macrophages through MHCII, SLAMF1, and co-stimulatory molecules (CD40, CD80, CD86) contributes to the control of intracellular *Mtb* growth [70,71,72]. Activated CD4 T cells secrete cytokines such as IL-2, IFN-γ, and TNF, which recruit other immune cells to the site of infection, promote differentiation of CD4 T cell subsets into effector cells, and activate macrophages. TNF promotes M1 macrophage activation and granuloma formation, and anti-TNF therapy for autoimmune disease is associated with an increased risk of TB reactivation [73,74]. IFN-γ enhances macrophage anti-mycobacterial activity by promoting autophagy, phagolysosomal fusion, delivery of ubiquitinated proteins to the lysosome, ROS production, and apoptosis [75]. Individuals with mutations in interferon gamma (*IFNG)* or its receptors develop disseminated infection with BCG and other non-tuberculous mycobacteria, and IFN-γ-deficient mice are highly susceptible to *Mtb* infection [76,77]. Although the magnitude of CD4 T cell-derived IFN-γ production does not consistently correlate with protection in humans, recent murine studies demonstrate that T cell-derived IFN-γ is required to limit lung and systemic *Mtb* burden [78], while PD-1 is needed to limit excessive IFN-γ production and prevent immunopathology [79]. CD4 T cells also provide IFN-γ and TNF-independent protective signals [79,80,81] that reprogram macrophage metabolism and polarization, limit necrotic lesion formation, and sustain CD8 T cell function [69,82,83,84]. Although the function of CD8+T cells in immunity against *Mtb* is not fully understood, multiple studies have demonstrated their protective role. For example, *Mtb*-infected CD8 T cell-deficient mice succumb, although not as rapidly as CD4 T cell-deficient mice. Cytotoxic CD8 T cells exert protection by directly interacting with infected macrophages and inducing apoptosis via perforins and granzymes [85]. Recently, specific peptide fragments of granzymes have been reported to inhibit *Mtb* replication within host cells in vitro [86]. Besides exerting cytotoxic effects, CD8 T cells produce cytokines IFN-γ, TNF, and IL-2, which activate immune cells to exert bactericidal effects and induce inflammation [85]. To conclude, CD4 T cells are central to protective immunity against *Mtb*, acting through both cytokine-dependent and cytokine-independent mechanisms to control bacterial growth, limit tissue damage, and sustain effective CD8 T cell responses; CD8 T cells contribute to complementary cytotoxic and cytokine-mediated mechanisms that enhance bacterial control.

### 3.2. B-Cell-Mediated Protection

Because *Mtb* can replicate intracellularly, antibodies have traditionally been viewed as ineffective at targeting and eliminating the bacteria. Additionally, patients with defects in antibody production and B cell defects, such as those with X-linked agammaglobulinemia or common variable immunodeficiency (CVID), do not show a clearly increased risk of TB infection, further contributing to the idea that humoral immunity is not particularly important in TB pathophysiology [87,88]. However, emerging evidence calls these assumptions into question. TB granulomas contain antibodies, plasma cells, and Ab-responsive Fc receptor (FcR)-bearing innate immune cells. In NHPs, animals with fewer B cells exhibit higher disease burden and worse outcomes at 4 and 12 weeks post-infection [89,90]. B cell deficiency in mice increases lesion bacterial burden and susceptibility to TB infection [91]. Furthermore, unbiased antibody profiling of the serum from individuals with latent TB (LTB) and active TB (ATB) revealed distinct humoral responses. Antibodies from individuals with LTBI showed unique Fc effector functional profiles and glycosylation patterns, and their antibodies enhanced human macrophage control of *Mtb* in vitro by promoting phagolysosomal fusion and inflammasome activation, compared with antibodies from people with active TB [92]. Abs against LAM enhance bacterial opsonization and restrict intracellular growth [93]. Furthermore, in BCG-primed NHPs, mucosal administration of a whole-cell, heat-killed vaccine strain (MTBVAC HK) induced PPD-specific mucosal immunoglobulins in the respiratory airways, resulting in the opsonization and phagocytosis of *Mtb* by macrophages, leading to enhanced bacterial control [94]. Interestingly, a post hoc analysis of the MVA85A vaccine (see Table 1) trial revealed that elevated Ag85A-specific IgG titers correlate with a reduced risk of TB disease [95], underscoring the growing evidence for a potential role for Abs in *Mtb*-protective immunity. Mechanistically, *Mtb*-specific antibodies can opsonize the bacilli, enhancing uptake via Fcγ receptors (FcγR) and complement receptors, and promoting phagolysosomal fusion and intracellular killing. Antibodies may also neutralize secreted virulence factors and cell wall components. Antibodies engage FcγR-expressing cells such as NK cells, neutrophils, and macrophages, triggering antibody-dependent cellular cytotoxicity (ADCC) and targeting infected cells for elimination, underscoring that humoral factors such as antibodies can reprogram various innate responses during TB infection [87,91,96,97,98]. Recently, it was found that immune complexes of *Mtb* with monoclonal antibodies targeting the *Mtb* protein PstS1 activate the NLRP3 inflammasome in macrophages and enhance IL-1β secretion. This antibody-mediated inflammasome activation is indispensable for early antibody-mediated protection in vivo and contributes to the efficacy of polyclonal sera from IV-BCG-immunized NHPs in conferring early protection against *Mtb* challenge [99]. In addition to antibody production, B cells may promote protection by presenting antigen, providing co-stimulatory signals to T cells, modulating T cell responses, and producing anti-inflammatory cytokines, such as IL-10, to prevent tissue damage [87,91,96]. Collectively, these findings support the idea that B cells and antibody-mediated effector functions likely contribute to control of *Mtb* infection and should be considered in vaccine design. Whether *Mtb* actively subverts the efficacy of B cell responses is not an area that has been investigated.

**Figure 2:**
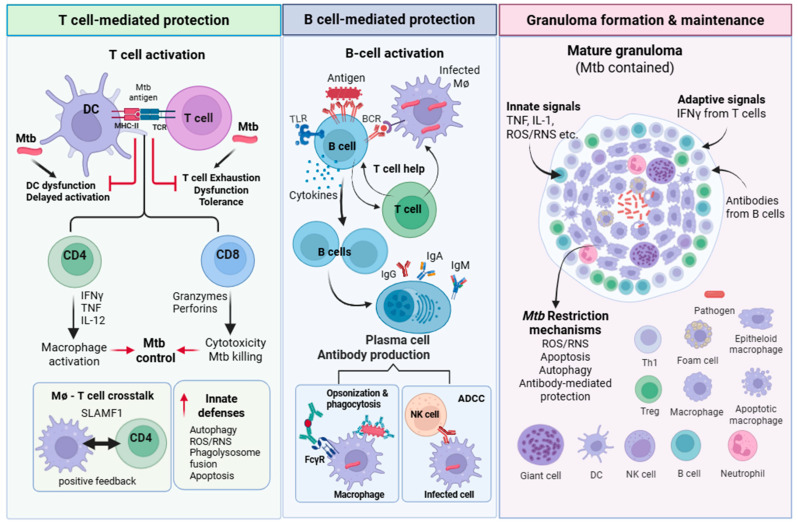
**Host-protective adaptive immune responses.** Antigen presentation by DCs to T cells primes antigen-specific T cells to drive T cell-mediated protection. Activated CD4 T cells produce crucial cytokines that activate macrophage effector functions, while CD8 T cells produce perforins and granzymes, which mediate cytotoxicity of infected cells. Optimal T cell-mediated protection depends on direct macrophage–T cell interactions, which induce SLAMF1 expression on infected macrophages and enhanced antimicrobial responses. B cells are activated by antigen binding, antigen presentation from infected macrophages, and T cell help. B cell activation drives plasma cell differentiation and antibody and cytokine production. Antibodies promote bacterial opsonization, enhance phagocytosis through FcγR receptor activation, and mediate antibody-dependent cell cytotoxicity (ADCC). Both T cell and B cell responses support the formation and maintenance of a structured granuloma. The granuloma features a central cellular or necrotic core containing Mtb, a surrounding layer of differentiated macrophages (foamy, epithelioid, multinucleated giant cells), distinct outer T cell and B cell zones, and plasma cells, encased by a fibrous capsule. Integrated innate and adaptive immune signals maintain granuloma structure to restrict Mtb replication and prevent bacterial dissemination. Created in BioRender. Mittal, E. (2026) https://BioRender.com/92lgdaj. Abbreviations: *Mtb*—*Mycobacterium tuberculosis*, DCs—dendritic cells, IMs—interstitial macrophages, NK—natural killer, ROS—reactive oxygen species, RNS—reactive nitrogen species, IFN—interferon, IL—interleukin, TNF—tumor necrosis factor, BCR—B cell receptor, TCR—T cell receptor, TLR—Toll-like receptors, SLAMF1—signaling lymphocytic activation molecule family member 1, FcγR—Fc gamma receptor, Ig—immunoglobulin, ADCC—antibody-dependent cell cytotoxicity, Th1—Type 1 T helper cells, Treg—regulatory T cells.

In conclusion, adaptive immunity against *Mtb* involves a coordinated immune response in which cellular immunity, mediated by T and B cells, and humoral immunity, orchestrated by antibodies produced by B cells, contribute to controlling bacterial infection in the lungs. While CD4 T cells play a central role in limiting bacterial growth, tissue damage, and dissemination via direct macrophage interactions and cytokine production, CD8 T cells play a complementary role in enhancing bacterial clearance through cytotoxicity and cytokine production. Although less well studied, B cells and antibodies play nuanced and potentially important roles in controlling *Mtb* infection by inducing bacterial opsonization, Fc receptor-mediated effector functions, ADCC, and inflammasome activation, and may be leveraged for enhanced vaccine efficacy. Adaptive immune cells work in coordination with innate immune cells to restrict *Mtb* replication and dissemination by forming a structured granuloma that can contain the bacteria within the granuloma, as shown in Figure 2.

## 4. Bacterial Evasion Mechanisms

**Figure 3:**
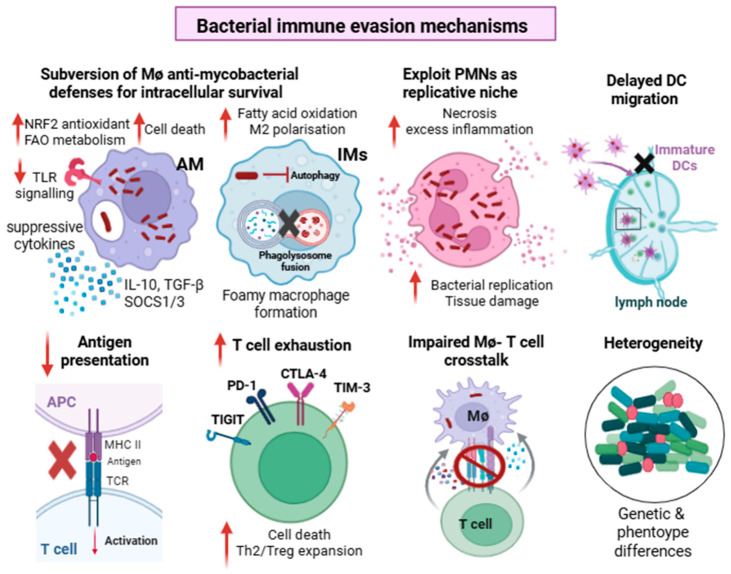
**Evasion strategies employed by *Mtb* to evade immune cell defenses**. *Mtb* employs multiple evasion mechanisms to survive intracellularly and cause disease. *Mtb* induces NRF2 antioxidant responses, suppresses TLR signaling, and exploits FAO metabolism to survive within AMs. In IMs/MDM, *Mtb* inhibits phagosome maturation and autophagy, reprograms macrophage metabolism from glycolysis to OXPHOS and FAO, and induces lipid accumulation and promotes foam cell formation. In neutrophils, *Mtb* promotes necrotic cell death, leading to excessive inflammation, tissue damage, and bacterial replication. *Mtb* delays DC trafficking from lung to the lymph nodes, impairing DC antigen presentation and T cell activation. Mtb suppresses T cell effector functions by inducing exhaustion, senescence, cell death and promotes differentiation of immunosuppressive Th2/Treg cells. *Mtb* impairs macrophage–T cell interaction by suppressing MHCII expression, antigen presentation, inducing an M2 phenotype and necrosis. Bacterial genetic and phenotypic diversity gives rise to subpopulations that differ in growth patterns, metabolism, virulence characteristics, drug susceptibility, immune tolerance and infection outcomes. Created in BioRender. Mittal, E. (2026) https://BioRender.com/92lgdaj.

Despite years of research, the failure to develop an effective vaccine is linked to our incomplete understanding of the multifaceted immune-evasion strategies employed by *Mtb* (Figure 3). The bacteria do not merely hide intracellularly; they systematically undermine host defenses, actively reprogram host cell biology, and interfere with cell–cell communication to create a permissive niche and establish chronic infection. Furthermore, bacterial heterogeneity in the host, ranging from actively replicating bacilli to dormant bacteria within biofilms and granulomas, poses further challenges for vaccine development. A CMV-vectored TB vaccine or BCG administered through a mucosal or intravascular route (rather than the conventional intradermal route) can generate substantially stronger, and sometimes sterilizing, immunity in NHPs [100,101,102]. Although these vaccine strategies are not clinically feasible, they demonstrate that protection from infection is biologically achievable. Similarly, the phase 2b study of the M72/AS01_E_ subunit vaccine (described in Table 1) showed that a therapeutic vaccine is also feasible, as it provided >50% efficacy in preventing TB disease in adults with latent *Mtb* infection [103]. This recent progress provides an opportunity to define correlates of protection to guide vaccine efforts. At the same time, understanding the molecular and cellular interactions that allow *Mtb* to subvert host immunity will also be critical to enabling novel vaccine strategies and overcoming the limitations of our current approaches.

### 4.1. Type VII Secretion Systems

*Mtb* possesses an arsenal of virulence factors, including proteins and lipids in the cell envelope and secreted effectors. *Mtb* encodes five specialized protein export systems known as type VII secretory systems (T7SS) or ESX (early secretory antigenic secretion) systems. ESX-1 is the most extensively studied and is recognized for its central role in virulence [104,105]. The ESX-1 system is encoded within the genomic region of difference 1 (RD1) locus, a region missing in BCG that accounts for its attenuation. ESX-1 secretes several substrates, including EsxA/ESAT-6 (early secreted antigenic target of 6 kDa) and EsxB/CFP-10 (culture filtrate protein of 10 kDa). ESX-1 enables *Mtb* to escape from the phagosome and enter the host cytosol by damaging the phagosomal membrane, a function attributed to pore formation by ESAT6 at acidic pH [106,107]. Phagosomal damage allows a plethora of other virulence factors to access the host cytosol (discussed below), perhaps explaining why the loss of ESX-1 is so profoundly attenuating. ESX-2 and -4 have also been implicated in phagosomal damage, while ESX-3 helps *Mtb* acquire iron and zinc, inhibits the ability of the host to repair phagosomal membrane damage, and blocks antigen presentation [104,108,109,110]. ESX-4 enables *Mtb* to secrete the tuberculosis-necrotizing toxin (TNT) into the cytosol, triggering necrotic cell death [111,112]. ESX-5 promotes the secretion of PE/PPE proteins and modulates host cell death and immunomodulation [104].

### 4.2. Evading and Disrupting Immune Recognition

*Mtb* has many PAMPs that are recognized by host PRRs and induce inflammation. However, *Mtb* employs a variety of strategies to subvert immune cell recognition. The complex mycobacterial cell envelope and capsule appear to shield underlying PAMPs in the cell wall [113]. The outer envelope lipid, phthiocerol dimycocerosate (PDIM), has been shown to intercalate into host membranes and hinder lipid raft organization, TLR signaling, and inflammatory responses. Phenolic glycolipids promote the recruitment of a permissive macrophage population and interfere with TLR4 signaling [114,115,116]. The *Mtb* protein PE_PGRS38 interacts with host ubiquitin ligase HAUSP, promoting degradation of TRAF6, a key intermediate in the TLR signaling pathway [117]. The *Mtb* serine hydrolase Hip1 inhibits TLR2- and TLR9-dependent macrophage activation and inflammatory cytokine production [118]. In addition, *Mtb* is thought to avoid the pro-inflammatory cascade associated with TLRs by engaging the mannose receptor (MR) and complement receptor 3 (CR3). This allows the bacilli a ‘silent’ entry that limits the activation of the NADPH oxidase, phagosome maturation, and pro-inflammatory cytokine production [119,120,121,122]. Collectively, these strategies may support the initial infection and persistence of the bacilli, whereas to promote transmission, the bacilli may benefit from driving a hyperinflammatory phenotype that causes tissue damage. One possibility is that *Mtb* alters cell envelope architecture and virulence gene expression to tune the host inflammatory response differently at different stages of infection.

### 4.3. Phagosomal Damage and Intracellular Survival

*Mtb* survives intracellularly by impairing phagosome maturation, inhibiting both non-canonical and canonical autophagy, and resisting acidic and oxidative defenses. Normally, a phagosome matures by successively acquiring Rab5 and Rab7 and fusing with the lysosome to become an acidic, degradative compartment. The *Mtb* protein NdkA inactivates host Rab GTPases, contributing to a block in phagosome maturation, while also impairing the NADPH oxidase [123]. The phosphatases SapM and PtpA dephosphorylate host phosphatidylinositol 3-phosphate (PI3P) and the proton pump V-ATPase, respectively, to prevent phagosome maturation [124,125]. In addition, the bacilli that are trafficked to the lysosome are relatively resistant to a low pH. The membrane protease MarP confers acid resistance and helps bacteria maintain intrabacterial pH [126,127]. *Mtb* expresses catalase (KatG), superoxide dismutase (SodA), and NuoG to detoxify free radical ROS and RNS generated by the host. CpsA, along with the virulence lipid PDIM and PPE/PE_PGRS proteins, impairs phagosomal recruitment of the NADPH oxidase, thereby interfering with non-canonical autophagy (LC3-associated phagocytosis) [128,129]. Both ESX1-secreted substrate EsxA and PDIM work synergistically to promote *Mtb* escape from the phagolysosome into the cytosol [130,131,132]. *Mtb* exposed to the cytosol recruits E3 ligases Parkin and Smurf2, which ubiquitinate *Mtb*. The autophagy adaptors (NDP52, p62, TAX1bp1) are then recruited, which link bacterial cargo to the autophagy machinery [63]. However, *Mtb* can impair autophagy and autophagosome maturation using multiple effector proteins, including PE/PPE proteins (PE_PGRS47, PPE51, PE_PGRS20), EIS, PknG, SapM, and PtpA [133]. These effectors enable *Mtb* to evade lysosomal killing despite activation of xenophagy. The ability of *Mtb* to impair lysosomal trafficking pathways impacts not just innate control, but also the ability of myeloid cells to present antigen and prime T cells, as discussed below. In addition, when activated T cells arrive in the lung, whether they are generated in the context of natural infection or vaccine-elicited immunity, their ability to drive bacterial clearance from the myeloid compartment depends upon overcoming these evasion strategies.

## 5. Challenges in Vaccine Design and Efficacy

### 5.1. Dendritic Cell Dysfunction and Delayed Activation of the Adaptive Immune Response

*Mtb* delays the onset of T cell responses by suppressing DC function, allowing *Mtb* to replicate largely unchecked until T cell responses are established in the lung [134]. In mice, it takes 8–11 days for *Mtb* to reach the draining lymph node, followed by an additional 5–10 days before antigen-specific T cell responses become detectable in the lung; altogether, it takes nearly 3 weeks before measurable adaptive immune responses are apparent in the lungs [134,135]. Under normal conditions, upon capturing antigens, DCs undergo maturation, upregulate the chemokine receptor CCR7, and migrate to draining lymph nodes, where they present processed antigens on MHC molecules in conjunction with co-stimulatory molecules (CD80, CD86, and CD40) to naive T cells. However, *Mtb* can interfere with multiple steps in this process. The bacterial Hip1 protein inhibits DC maturation, reduces MHCII and co-stimulatory molecule surface expression, limits antigen presentation, and suppresses Th1-inducing cytokine production [136]. *Mtb* impedes DC migration to the lymph node by downregulating integrins (CD11a, CD11b, CD18), impairing their adherence to lung endothelial cells [137]. *Mtb* also engages the DC immunoadaptor DAP12 (DNAX-activating protein of 12 kb), which induces the expression of the negative regulator IRAK-M, increases IL-10 production, and blunts Th1 cell activation [138]. *Mtb*-secreted glycoprotein Rv1016c inhibits DC maturation, co-stimulatory molecule expression, and cytokine production by targeting the TLR2/STAT/SOCS3 signaling pathway. It also impairs DCs from polarizing naïve T cells to Th1 and Th17 cells [139]. The *Mtb* virulence factors PE_PGRS47 and PPE51 inhibit autophagy in DCs, impairing the degradation and processing of *Mtb* [140,141]. In addition, by blocking apoptosis in infected neutrophils, the *Mtb* protein NuoG impairs antigen acquisition by myeloid DCs and delays activation of antigen-specific T cells [142]. In addition to delaying T cell priming, the multiple ways in which *Mtb* impair DC function may limit the pool of *Mtb* epitopes available for antigen presentation, thereby reducing the diversity and magnitude of T cell responses. *Mtb* also enhances IL-10 production by DCs, promoting a tolerogenic phenotype and impairing Th1 responses [143]. The interaction of manLAM in the *Mtb* envelope with the C-type lectin receptor, DC-SIGN, may further impair DC maturation and co-stimulatory ligand (CD80, CD86) expression, while promoting IL-10 production [144,145]. Experimental restoration of DC function by targeting CD103+ DCs or CD40-40L pathways accelerates the accumulation of protective CD4 T cells and improves *Mtb* control [146], underscoring that pathogen-imposed DC dysfunction can be overcome. Overall, these findings suggest that even if a vaccine induces a high-quality protective T cell response, the intrinsic delay in T cell priming, expansion, and homing to the lungs remains a major barrier; therefore, a preventative vaccine may need to establish robust tissue-resident memory T cell populations poised to respond quickly in the lung. Recent studies examining how IV BCG protects NHPs identified Th1/Th17 cytokine-producing *Mtb*-specific CD4 T cells, innate CD8α+ lymphocytes, cytotoxic Vδ1/3 γδ T cells, and cytolytic CD69-granzyme B+ NK cells as important for protection [147,148,149].

### 5.2. T Cell Exhaustion, Dysfunction, and Establishment of Immune Tolerance

Although T cells play an important role in protective responses during *Mtb* infection, they fail to reliably provide sterilizing immunity. During chronic infection, *Mtb*-specific T cells undergo progressive dysfunction and exhaustion, leading to impaired activation, cytokine production, and effector functions, while checkpoint pathways contribute to immune tolerance [150,151]. *Mtb* actively suppresses protective Th1 responses and Th17 differentiation [152,153,154]. Immune escape through antigenic variation and diversifying selection in *Mtb* is relatively rare, as most known human T cell epitopes appear to be highly conserved. However, a few dominant antigens exhibit genetic variation in clinical isolates. A notable example is the *esxH* gene (encoding the immunodominant antigen TB10.4). A naturally occurring A10T polymorphism in the *esxH* gene alters the responses of immunodominant TB10.4-specific CD8 T cells, affecting their priming and ability to recognize infected macrophages [155]. Furthermore, the evolving transcriptional regulation of virulence and antigen genes, including reduced expression of EsxA and EsxB in highly transmissible and drug-resistant strains, may further reduce the presentation of canonical T cell epitopes, allowing the bacteria to evade T cell-mediated immune responses. Together, polymorphisms and transcriptional regulation of immunodominant antigens may lead to non-protective T cell responses and create a potential mismatch between vaccine-induced T cells and antigens presented by infected cells [156].

*Mtb* further subverts T cell immunity by diminishing Th17 responses while promoting regulatory, exhausted, and anergic T cell states. Individuals with active TB have fewer circulating Th17 cells than healthy individuals and individuals with latent TB, which may be related to reduced IL-6R expression on CD4 T cells [157]. In addition, ESX-1 and PDIM suppress IL-23 production by lymph node-resident DCs, thereby impeding Th17 differentiation [152]. *Mtb* also promotes the expansion of Foxp3+ regulatory T cells (T_regs_) by inducing a cytokine milieu that favors Treg development and maintenance. By expressing high-affinity CD25 receptor, Tregs deplete available IL-2, a cytokine crucial for effector T cell proliferation and survival. Additionally, Tregs induce apoptosis in effector T cells by secreting granzymes and perforins [154,158]. *Mtb* drives T cell exhaustion through chronic antigenic stimulation, mitochondrial dysfunction, metabolic reprogramming, upregulation of inhibitory receptors (PD-1, Tim-3, LAG-3, CTLA-4, and CD160), and reduced production of IL-2, TNF, and IFN-γ [151,159,160]. *Mtb* may also induce T cell anergy by disrupting phosphorylation of proteins involved in TCR signaling (ZAP-70, MAPK, and TCRζ) [161]. Anergic T cells fail to respond to antigen, and they suppress Th1 responses through IL-10 production [162]. Constitutive expression of dominant T cell antigens, such as ESAT6, throughout the infection drives CD4 T cells towards functional exhaustion. Moreover, prolonged antigen exposure drives T cells into a highly polarized (KLRG1+ CX3CR1+) state, and they remain localized in the vasculature rather than entering the lung parenchyma [163,164]. Together, these processes divert CD4 T cells away from durable, lung-homing, polyfunctional effector states, a pattern that therapeutic TB vaccines will need to counteract.

### 5.3. Disruption of Macrophage–T Cell Crosstalk

Cognate interactions between macrophages and T cells are mediated by the engagement of the TCR with peptide–MHC complexes on the macrophage surface. CD4 T cells reduce intracellular *Mtb* burden in MHCII-expressing macrophages more effectively than in MHCII-deficient macrophages, demonstrating that direct recognition of infected macrophages by T cells through TCR–peptide–MHCII engagement is crucial for *Mtb* control [71]. Direct cognate interactions enhance glycolysis, IFN-γ-mediated nitric oxide production, and other antimicrobial programs in macrophages [165]. We recently showed that direct cell–cell contact between *Mtb*-specific CD4+ T cells and infected macrophages induces SLAMF1/CD150 expression on infected macrophages. SLAMF1 subsequently enhances ROS production and bacterial control [70]. However, *Mtb* has mechanisms that disrupt effective macrophage–T cell interactions. Effector CD4 and CD8 T cells differ in their ability to interact with infected macrophages, with CD8 T cells showing very limited ability to recognize infected macrophages [166]. In mice, only a fraction of lung *Mtb*-specific CD4 and CD8 T cells efficiently recognize *Mtb*-infected macrophages at low multiplicity of infection (MOI), indicating that many antigen-specific T cell clones fail to detect infected target cells [167] and suggesting that impaired macrophage antigen presentation limits T cell detection of infected targets. Robust CD8 T cell activation requires a high bacterial burden together with ESX-1-dependent antigen export and cross-presentation of *Mtb* antigens by infected macrophages [168]. In addition, kinesin 2-mediated vesicular export of bacterial proteins from infected macrophages diverts antigens away from the MHCII presentation pathway in the infected cells, and some antigens may serve as decoys, resulting in the suboptimal CD4 T cell activation [155,169]. *Mtb* also promotes rapid, type I IFN-driven neutrophil infiltration into the lungs, which interferes with the ability of T cells to interact with macrophages [170,171]. *Mtb* may suppress MHCII expression in some macrophage populations. For example, during early infection, NRF2 activation in AMs inhibits CIITA, a master transcription factor for MHCII expression, and prevents activation of antigen-specific T cells, despite exogenous IFNγ or PAMP stimulation [172]. With the onset of T cell immunity, *Mtb* antigen-specific T cells more efficiently clear *Mtb* in AMs than IMs, indicating that *Mtb* in IMs may be refractory to vaccine-enhanced T cell-mediated control [167,173]. In chronic infection, *Mtb* downregulates the expression of key antigens, such as Ag85B, suppresses the activation and maintenance of polyfunctional antigen-specific T cells [163,174]. *Mtb* exports its glycolipid manLAM directly to the CD4 T cell membrane to inhibit Lck phosphorylation and TCR signaling and disrupts immune synapse formation between macrophages and T cells [161]. Human data similarly suggest that macrophage heterogeneity creates niches that are poorly recognized by T cells, as memory CD4 T cells from LTBI donors readily respond to *Mtb*-infected GM-CSF-differentiated (M1-like) macrophages but not to M-CSF-differentiated (M2-like) macrophages [175]. This highlights the need for vaccines that program T cells to recognize and respond to infected macrophages across diverse polarization states. Furthermore, in granulomas, T cells are sequestered in peripheral lymphoid cuffs, physically separated from the myeloid cells harboring bacteria in the granuloma core [176,177]. Additionally, macrophages near the necrotic core express high levels of indoleamine 2,3-dioxygenase (IDO), depleting tryptophan and generating kynurenines that inhibit T cell proliferation and function [178,179,180]. Therefore, even if a therapeutic vaccine induces protective T cells, the granuloma structure and microenvironment may prevent them from trafficking to the lesion core or render them ineffective at the local site of infection. Together, these mechanisms uncouple antigen-specific T cell responses from effective recognition of infected macrophages, thereby undermining T cell-mediated control of *Mtb*, whether induced by vaccination or natural infection. This systematic disruption of macrophage–T cell crosstalk by *Mtb* may help explain why the vaccine candidate, MVA85A, failed despite robust Ag85-specific IFN-γ+ CD4 T cell responses. Using markers such as SLAMF1 expression on macrophages to monitor macrophage–T cell interactions may help assess the success of approaches designed to enhance these interactions. Overcoming these barriers will likely require multipronged strategies. For example, inhibiting the immunosuppressive enzyme IDO1 in combination with vaccination may enhance the ability of a therapeutic vaccine to clear bacilli.

### 5.4. Bacterial Heterogeneity

An effective TB vaccine must contend with extensive *Mtb* genetic and phenotypic heterogeneity. There are ten human-adapted lineages in the *Mtb* complex, each with unique single-nucleotide polymorphisms (SNPs), genomic deletions, geographic associations, and virulence and transmission characteristics [6,181,182]. For example, strains of lineages 2 and 4 exhibit enhanced transmission; lineage 2 strains exhibit higher mutation rates and increased development of multi-drug resistance (MDR); and L1 strains have been associated with increased risk of cavitary disease and osteomyelitis compared with modern lineages (L2-4). A genome-to-genome (g2g) analysis of human and *Mtb* genomes of TB patients in Peru identified an intronic SNP (rs3130660-A) in the human FLOT1 gene that predisposes to infection with an L2 subclade *Mtb* strain [183], indicating that both host and bacterial genetic traits modulate disease risk. Studies on the phenotyping of *Mtb* clinical isolates using a molecular barcoding strategy have shown that lineage 2 (mL2) strains are more resistant to BCG-induced immune protection [184]. Thus, vaccines optimized to target a particular bacterial genotype or phenotype may show reduced effectiveness when tested against diverse clinical isolates. In addition, studies in Diversity Outbred (DO) mice have shown that host genetic background influences the immune response to BCG vaccination, with some mice mounting a protective Th1 or Th17 response and others mounting a non-protective Th2 response, highlighting that host diversity also influences vaccine efficacy [185,186].

Beyond *Mtb* lineage-level diversity, clonal *Mtb* populations display significant phenotypic heterogeneity in growth rates, metabolic profiles, drug susceptibilities, and stress responses driven by variation in rRNA expression, partitioning of cellular components during division, and cell size [187]. Under stress conditions, this phenotypic heterogeneity is amplified, by the emergence of subpopulations of non-growing, metabolically active persisters and antibiotic-tolerant variants that facilitate *Mtb* survival [188]. Within the host, *Mtb* encounters spatiotemporally heterogeneous environments; for example, *Mtb*it exploits macrophage metabolic diversity to evolve into redox-diverse subpopulations, with OXPHOS-high macrophages harboring drug-tolerant *Mtb* in a reduced redox state and glycolytic macrophages harboring drug-susceptible, redox-stressed *Mtb* [189]. In humans and NHPs, individual lungs contain multiple granulomas that differ in morphology (necrotizing, fibrotic, or cavitary), cellular composition/organization, cytokine milieu, and sterilizing capacity [190,191]. Granulomas establish gradients in oxygen, pH, nutrient availability, immune cell composition, and drug penetration, creating distinct micro-niches that permit actively replicating or quiescent persister bacilli to coexist [176,192,193]. The identification of multiple coexisting *Mtb* subclones associated with lesions exhibiting disparate antibiotic response kinetics in an MDR-TB patient corroborates that granuloma diversity promotes bacterial heterogeneity [192,194]. Thus, an effective vaccine may need to drive protective responses targeting both replicating bacilli with distinct phenotypes and non-replicating bacteria. In addition, *Mtb* dramatically remodels its antigen expression profile as it adapts to different microenvironments and stages of infection. For example, Ag85B is highly expressed during rapid aerobic replication but downregulated during chronic infection and hypoxia [163,174,195]. Therefore, a vaccine designed to induce T cells specific for Ag85B may fail to target bacterial populations residing in different microenvironments. This has important implications for vaccines that aim to protect against both infection and disease progression across infection stages and lesion microenvironments. Combining antigens expressed early by replicating bacilli (e.g., Ag85B, ESAT-6, TB10.4, and Mtb8.4), during resuscitation (RpfE), and during latency (Rv2660c, Rv2628, and HspX) may be necessary to confer protection against active, chronic and latent infection [196,197,198,199]. A recent study used systematic antigen mining, together with a trivalent mRNA vaccine platform, to identify protective antigens. They found substantial heterogeneity in the protective efficacy of different antigens, with a combination of subdominant antigens outperformed BCG in mice. In addition, antigen immunogenicity did not reliably predict protective efficacy [200]. Overall, the heterogeneity of the bacilli complicates identifying ideal antigens, and future vaccine efforts should aim to simultaneously target multiple *Mtb* subpopulations. A complementary approach would be to reduce bacterial heterogeneity, for example, by combining a therapeutic vaccine with host-directed therapies that modify granuloma architecture or the bacterial niche.

### 5.5. Rational TB Vaccine Design: Engaging Protective Innate and Adaptive Immunity

The ‘End TB’ strategy, developed by the World Health Organization to eliminate the TB epidemic by 2035, aims to reduce TB incidence by 90% and mortality rates by 95%. Vaccines are critical to that mission. Currently, about 17 vaccine candidates are in clinical development, including 6 that are in phase 3 clinical trials [201]. Vaccine candidates, together with their potential mechanisms to generate protection against *Mtb*, are listed in Table 1. These vaccines use different platforms, including recombinant protein-based vaccines (M72/AS01E; GaMtbvac), live-attenuated vaccines engineered in *Mtb* (MTBVAC), and modified BCG vaccines (VPM1002, AERAS-422). They aim to induce protective immunity by eliciting *Mtb*-specific, polyfunctional CD4+ and CD8+ T cells, tissue-resident memory T cells localized in the lung parenchyma, and antibody-mediated immunity through Fcγ receptor-dependent phagocytosis [96,202,203,204,205,206,207]. However, these strategies, which rely on adaptive immunity, may not adequately address the immune dysfunction caused by *Mtb*. Addressing the current challenges in vaccine design requires harnessing the known protective innate and adaptive responses during *Mtb* infection while countering the complex evasion mechanisms *Mtb* uses to disrupt their crosstalk. By impairing innate immunity and reshaping granuloma architecture, *Mtb* creates microenvironments that limit optimal T and B cell priming and restrict the access of effector cells and antibodies to bacilli. This highlights the need for vaccines that simultaneously engage multiple innate pathways and provide protection at different infection stages during disease progression. Vaccines, adjuvants and host-directed treatments that shift macrophages towards more bactericidal phenotypes are likely to enhance vaccine-induced T cell responses and protection. Approaches that promote both IL-12-rich and protective Th1/Th17-skewed responses and induce trained immunity in myeloid cells enabling them to mount rapid bactericidal responses during early infection, should be prioritized. Innate immune cells can undergo long-term functional reprogramming through trained immunity, which involves long-lasting epigenetic modifications, metabolic reprogramming toward glycolysis, elevated expression of pattern recognition receptors (particularly TLR2/TLR4), and increased production of inflammatory cytokines (TNF-α, IL-1β, IL-8) upon restimulation with microbial pathogens. Future vaccines could train innate immune cells to establish a first line of defense, while also eliciting adaptive responses. Considering a macrophage-centric approach, it maybe possible to reprogram AMs to favor glycolysis and M1 polarization, block *Mtb*-driven suppression of autophagy and antigen presentation, or harness CD38 expression, all of which are associated with improvedbacterial control [165,208]. With a better fundamental understanding of immune responses and bacterial virulence, it may be possible to establish sterilizing immunity against *Mtb* by combining metabolic and immunological reprogramming strategies, harnessing innate and adaptive immunity, and overcoming *Mtb* immune evasion mechanisms.

Optimizing adaptive immune mechanisms for TB vaccines requires antigen choice and platforms that elicit the full spectrum of protective T cell and B cell functions in vivo and overcome *Mtb*-induced dendritic cell dysfunction, impaired migration, and reduced co-stimulation, which otherwise weaken the bridge between innate and adaptive immunity. The presence of antibodies with distinct Fc effector profiles and differential capacity to enhance macrophage control in individuals with LTBI underscores the need to prioritize antigens and platforms that not only drive strong immunogenicity but also optimize Fc-mediated antibody functions for durable control. Eliciting antibodies that neutralize critical virulence factors is one approach to overcome immune evasion that has not been deeply explored. Combining antigens expressed at different stages of infection can help ensure that vaccine-induced responses target both replicating and non-replicating bacilli across heterogeneous lesions in active and latent TB. Future vaccine strategies should focus on platforms and delivery routes that establish long-lasting lung-resident memory T and B cells, promote protective granuloma organization, and enhance interactions between activated myeloid and lymphoid cells in the lung and draining lymphoid nodes, while targeting antigens less prone to downregulation. Overall, an effective TB vaccine strategy must explicitly integrate innate and adaptive immunity and address *Mtb’s* specific immune evasion tactics, thereby translating mechanistic immunological insights into practical design principles for next-generation vaccines.

## 6. Conclusions

Great progress has been made recently in our understanding of *Mtb* pathogenesis and host immune responses. However, developing an effective TB vaccine has proven challenging. The absence of validated immunological correlates of protection, the limited availability of animal models that accurately recapitulate the complex pathology of human granulomas, and the immune evasion mechanisms adopted by *Mtb* create barriers to vaccine development efforts. The failure of the MVA85A vaccine indicates that traditional approaches focused on boosting T cell responses are inadequate [209]. The protective immunity observed in animal models underscores that sterilizing immunity is biologically achievable. The emergence of multi-omic approaches, single-cell technologies, and artificial intelligence all have the potential to drive vaccine development forward. The future of effective vaccine development may depend on combinatorial strategies that jointly address bacteria-, host-, and tissue-level complexity. These include targeting antigens expressed across multiple bacterial physiological states, establishing lung-resident T cell memory, considering B cell-mediated protection, optimizing vaccine routes, and exploiting trained immunity to modulate the physiology of innate immune cells.

**Table 1:** TB vaccine candidates in clinical development and their immunological mechanisms of protection against *Mycobacterium tuberculosis*.

Vaccine Type	Vaccine Candidate (Composition)	Mode of Action/Key Immunological Mechanisms	References
Live attenuated whole-cell vaccines	VPM1002 (recombinant BCG (ΔureC::hly) urease C-deficient strain expressing listeriolysin O)	Promotes phagosome acidification, phagolysosomal fusion, and phagosome membrane perturbation and release of mycobacterial antigens into the cytosol. Promotes autophagy, apoptosis, and inflammasome activation. Enhances antigen presentation and robust priming of CD4 and CD8 T cells. Overcomes BCG’s limitations of poor cytosolic antigen access and suboptimal CD8 priming by mimicking the effects of ESX-1 activity. Induces both type 1 and Type 17 cytokine responses to confer enhanced protection.	[210,211,212,213,214]
	MTBVAC (*M. tuberculosis* with deletion mutations in two virulence genes *phoP*, *fadD26.* It contains the RD1 locus)	Induces broader, specific Th1 CD4 T cell immunity than BCG, as it retains the RD1 locus with more T cell epitopes of *Mtb*. Generates trained immunity through the induction of glycolysis, glutaminolysis, and histone methylation of pro-inflammatory cytokine genes. Potentially reduces *Mtb* immune escape by presenting a wider epitope repertoire. May enhance early innate control despite *Mtb*’s suppression of PRR and cytokine signaling through trained immunity.	[215,216,217]
	BCG (revaccination, travel vaccine) *(M. bovis* BCG Tokyo-172 strain)	Boosts BCG-primed T cell responses, trained immunity, and cytokine production upon heterologous stimulation. Increases IFNγ and TNF-producing *Mtb*-specific CD4 T cells. Overcomes waning BCG-induced protection and *Mtb*-driven innate hypo-responsiveness through trained immunity in monocytes/NK cells, enhances Th1 responses and may increase early containment.	[218,219,220,221]
Inactivated whole-cell vaccines	DAR-901 heat-inactivated *Mycobacterium obuense*	Stimulates cellular and humoral immunity. Induces strong IFNγ responses to both DAR-901 and *Mtb* antigen preparations. Designed to boost BCG immunity against tuberculosis.	[222,223,224]
	ImmuVac heat-killed *Mycobacterium indicus pranii*	Potent activator of innate and adaptive immunity inducing Th1 and Th17 responses, macrophage and DC activation. Promotes secretion of IL-1β and hBD-2. Shares epitopes with *Mtb* and activates antigen-specific polyfunctional T cells. May help overcome and *Mtb* induced Th2 skewing and Treg cell response by enhancing Th1/Th17 polarization.	[225,226,227]
	RUTI (polyantigenic liposomal suspension of detoxified *M. tuberculosis* cell wall components	Induces strong polyantigenic cellular immunity through activation of IFNγ+ CD4 and CD8 T cells against specific *Mtb* antigens (PPD, ESAT-6, HSP16.3, Ag85B, PsTS1, etc). Reduces bacillary load and pulmonary granulomatous infiltration in murine and guinea pig *Mtb* infection models. Designed to boost Th1 responses and reduce bacterial burden in animal models of LTBI. Increases the frequency of non-classical Ly6C-monocytes to promote cell-mediated response and limit excessive inflammation.	[228,229,230]
Subunit vaccine	M72/AS01E recombinant fusion protein M72 composed of *Mtb* proteins Mtb32A, Mtb39A and AS01E adjuvant	Induces potent polyfunctional M72-specific CD4 T cells which can persist for ≥3 years. AS01E enhances DC activation and antigen presentation. Enhances Th1 responses in latently infected adults, boosting CD4 responses. Protects against progression from LTBI to active pulmonary disease.	[103,207,231,232,233]
	GamTBMtbvac (Ag85A and ESAT6-CFP10 fusion proteins fused with dextran-binding domain from *Leuconostoc mesenteroides* and DEAE-dextran/CpG adjuvant)	Induces Ag85A, ESAT6-CFP10-specific IFNγ-producing CD4+ T cells proliferation and activation. CpG/DEAE-dextran drives Th1-skewed responses via TLR9 and enhanced DC activation. Utilizes highly immunodominant *Mtb* antigens to strengthen Th1 immunity and may prevent the risk of T cell exhaustion or dysfunction. CpG-driven innate activation may counter *Mtb*-mediated suppression of TLR signaling, thereby supporting better early bacterial control.	[233,234,235,236]
	ID93+GLA-SE (recombinant fusion protein ID93 made of four tandem-linked *Mtb* antigens Rv3619, Rv1813, Rv3620, Rv2608 and TLR4 agonist adjuvant GLA-SE)	Targets antigens expressed across active and latent disease stages to strengthen T cell responses. Induces polyfunctional antigen-specific CD4 T cell activation and antibody production. TLR4-driven Th1 skewing and functional antibodies generated by the GLA-SE adjuvant may overcome suboptimal opsonization and phagocytic killing, and increase cytokine production.	[233,237,238,239]
	H107e/CAF10b (eight *Mtb* antigens formulated with a novel liposomal adjuvant CAF10b containing MINCLE and TLR9 agonists)	Co-administration with BCG enhances the immunogenicity and protective effects of BCG. Increases the clonal diversity of the CD4 T cell repertoire induced by BCG, establishes long-lived immunity. CAF10b adjuvant drives strong Th1 and Th17 responses. *Mtb*-specific antigens and strong Th17/Th1 responses induced by the vaccine may enhance mucosal protection and early recruitment of neutrophils and T cells to the lungs, potentially overcoming BCG’s limited efficacy in adults.	[205,233,240,241,242]
	AEC/BC02 (Ag85b, ESAT6-CFP10 fusion proteins and BC02 adjuvant composed of BCG-CpG DNA and aluminum hydroxide)	Induces a higher frequency of antigen-specific Ifnγ-secreting T cells. Vaccination combined with chemotherapy reduces bacillary load and lowers drug-resistance development. CpG DNA (BC01) activates macrophages via TL9-NF-κB and MAPK signaling, and upregulates the expression of MHCII and co-stimulatory molecules. Highly immunogenic antigens combined with CpG/alum adjuvant may drive protective Th1-biased cellular responses, improve antigen presentation and cytokine production, and boost antigen-specific antibody production.	[233,243,244,245,246]
Viral vector-based vaccine	AdHu5Ag85A (human adenovirus serotype-5 vector-expressing Ag85A)	Aerosol delivery of the vaccine induced robust Ag85-specific CD4 and CD8 T cells, with Ifnγ, TNF, and IL-2 production in the airways, and established tissue-resident memory, compared with intramuscular immunization. Vaccine-induced tissue-resident memory T cell responses can rapidly induce IFNγ secretion at the sites of *Mtb* infection in the airways, enhance the early activation of macrophages, and may limit intracellular survival of *Mtb*.	[247,248,249,250]
	Ad5-105K (adenovirus type 5 vector-expressing Ag85A, Mtb32A and Mtb39A)	Formulated as a liquid for nebulized aerosol inhalation by mouth. Designed to induce both mucosal immunity in the respiratory tract and systemic immune response against *Mtb*. Induces polyfunctional antigen-specific CD4 and CD8 T cells responses in the respiratory mucosa. Similar to AdHu5Ag85A, the Ad5 virus capsid proteins may activate innate immunity via TLR-dependent pathways, enhance antigen presentation and cytokine production, and induce trained immunity in alveolar macrophages to rapidly control *Mtb* growth at the initial site of infection. May target bacilli during dormancy or reactivation phases, similar to M72 vaccine (Mtb32A and Mtb39A) that showed 50% efficacy against pulmonary TB in LTBI patients.	[207,247,248,251]
	ChAdOx1.85A+MVA85A (simian adenoviral vector expressing Ag85A administered by aerosol route followed by intramuscular administration of modified vaccinia Ankara virus expressing Ag85A)	Induces robust polyfunctional Ag85A-specific CD4 and CD8 T systemic cellular and humoral immune responses. Combination immunization with a prime-boost strategy significantly enhanced the frequency of antigen-specific T cells and IFNγ production in BCG-vaccinated healthy adults. Aims to circumvent BCG’s limited ability to induce CD8 responses and waning immunity by boosting cellular immunity against immunodominant *Mtb* antigen. May enhance homing of antigen-specific T cells to the lungs, early intracellular bacterial killing and bacterial control	[252,253,254]
	TB/FLU-05E (recombinant attenuated influenza vector Flu/THSP co-expressing truncated NS1 protein and full-length *Mtb* proteins TB10.4 and HspX)	Intranasally delivered influenza vector induced robust tissue-resident T cells specific for TB10.4 and HspX antigens, as well as antibody production in the respiratory mucosa. Aims to enhance *Mtb* clearance early during infection by localizing tissue-resident polyfunctional T cells, mucosal antibodies near the site of entry, and overcoming *Mtb*-mediated local immune suppression.	[202,255,256,257,258,259]
DNA/RNA vaccines	BNT164a1 and BNT164b1 (mRNA vaccines encoding eight *Mtb* antigens: Ag85A, Hrp1, ESAT-6, RpfD, RpfA, HbhA, M72, VapB47). BNT164a1 uses unmodified mRNA and BNT164b1 uses N1-methylpsuedouridine mRNA	Vaccine encodes *Mtb* antigens expressed across different infection stages In mice, prime-boost immunization elicited antibody and T cell responses against all eight *Mtb* antigens and reduced bacterial burden of multiple *Mtb* strains. Vaccine-induced protection correlated with granuloma infiltration by CD8 T cells with memory precursor phenotype. Aims to enhance antigen presentation, innate sensing and induction of robust CD4 and CD8 T cell responses targeting antigens expressed during early, chronic or latency infection stages. By broadening the epitope coverage, this vaccine may limit bacterial escape from host immune recognition.	[260,261,262]

## Data Availability

No new data were created or analyzed in this study.

## Abbreviations

The following abbreviations are used in this manuscript:

CCR	C-C chemokine receptor
CD80/86	Cluster of differentiation 80/86
cGAS	Cyclic GMP-AMP synthase
CIITA	Class II major histocompatibility complex transactivator
CMV	Cytomegalovirus
CTLA-4	Cytotoxic T-lymphocyte-associated protein 4
CVID	Common variable immunodeficiency
DO	Diversity Outbred
ESX-1	Early secretory antigenic target 6 kDa system 1
FcγR	Fc gamma receptor
FAO	Fatty acid oxidation
HAUSP	Herpesvirus-associated ubiquitin-specific protease
IFN	Interferon
IL	Interleukin
KLRG1	Killer cell lectin-like receptor G1
LAG-3	Lymphocyte-activation gene 3
LAM	Lipoarabinomannan
LC3	Microtubule-associated protein 1A/1B-Light Chain 3
LTBI	Latent TB infection
MAPK	Mitogen-activated protein kinase
MHCII	Major Histocompatibility Complex (MHC) II
MOI	Multiplicity of infection
*Mtb*	*Mycobacterium tuberculosis*
*MTB*C	*Mycobacterium tuberculosis* complex
NADPH	Nicotinamide adenine dinucleotide phosphate
NDP52	Nuclear dot protein 52 kDa
NF-κB	Nuclear factor kappa-light chain enhancer of activated B cells
NHP	Non-human primate
NO	Nitric oxide
OXPHOS	Oxidative phosphorylation
PD-1	Programmed cell death protein 1
PDIM	Phthiocerol dimycocerosate
PE protein	Proline–glutamic acid
PPE	Proline–proline–glutamic acid protein
PstS1	Phosphate-binding protein S1
Rab5/7	Ras-related protein Rab-5a/7a
SLAMF1	Signaling lymphocyte activation molecule family member 1
SNPs	Single-nucleotide polymorphisms
STING	Stimulator of interferon genes
TAX1BP1	Tax1 (human T cell leukemia virus type 1)-binding protein 1
TCR	T cell receptor
TGF-β	Transforming growth factor-beta
Tim-3	T cell immunoglobulin and mucin domain-containing protein-3
TNF	Tumor necrosis factor
TRAF6	TNF receptor-associated factor 6
